# Association Between SLC30A8 rs13266634 Polymorphism and Risk of T2DM and IGR in Chinese Population: A Systematic Review and Meta-Analysis

**DOI:** 10.3389/fendo.2018.00564

**Published:** 2018-09-25

**Authors:** Fang Dong, Bao-huan Zhang, Shao-ling Zheng, Xiu-xia Huang, Xiu-ben Du, Ke-hui Zhu, Xiao-jing Chen, Jing Wu, Dan-dan Liu, Zi-hao Wen, Xiao-qian Zou, Yu-mei Liu, Shi-rui Dong, Fang-fang Zeng, Guang Yang, Chun-xia Jing

**Affiliations:** ^1^Department of Epidemiology, School of Medicine, Jinan University, Guangzhou, China; ^2^Department of Information and Statistics, Shenzhen Hospital of Guangzhou University of Chinese Medicine, Shenzhen, China; ^3^Department of Pathogenic Biology, School of Medicine, Jinan University, Guangzhou, China; ^4^Guangzhou Key Laboratory of Environmental Exposure and Health, Guangdong Key Laboratory of Environmental Pollution and Health, Jinan University, Guangzhou, China

**Keywords:** type 2 diabetes mellitus, impaired glucose regulation, SLC30A8, rs13266634, polymorphism, meta-analysis

## Abstract

**Introduction:** Published data regarding the association between solute carrier family 30, member 8 (*SLC30A8*) rs13266634 polymorphism and type 2 diabetes mellitus (T2DM) and impaired glucose regulation (IGR) risks in Chinese population are in-consistent. The purpose of this meta-analysis was to evaluate the association between *SLC30A8* rs13266634 and T2DM/IGR in a Chinese population.

**Material and Methods:** Three English (PubMed, Embase, and Web of Science) and three Chinese databases (Wanfang, CNKI, and CBMD database) were used for searching articles from January 2005 to January 2018. Odds ratio (OR) and 95% confidence interval (95%CI) were calculated with the random-effect model. Trial sequential analysis was also utilized.

**Results:** Twenty-eight case-control studies with 25,912 cases and 26,975 controls were included for SLC30A8 and T2DM. Pooled risk allele C frequency for rs13266634 was 60.6% (95%CI: 59.2–62.0%) in the T2DM group and 54.8% (95%CI: 53.2–56.4%) in the control group which had estimated OR of 1.23 (95%CI: 1.17–1.28). Individuals who carried major homozygous CC and heterozygous CT genotype were at 1.51 and 1.23 times higher risk of T2DM, respectively, than those carrying minor homozygous TT. The most appropriate genetic analysis model was the co-dominant model based on comparison of OR1, OR2 and OR3. Five articles that involved 4,627 cases and 6,166 controls were included for SLC30A8 and IGR. However, no association was found between SLC30A8 rs13266634 and IGR (C vs. T, OR = 1.13, 95%CI: 0.98–1.30, *p* = 0.082). TSA revealed that the pooled sample sizes of T2DM exceeded the estimated required information size but not the IGR.

**Conclusion:** The present meta-analysis demonstrated that *SLC30A8* rs13266634 was a potential risk factor for T2DM, and more studies should be performed to confirm the association between rs13266634 polymorphism and IGR.

## Introduction

Type 2 diabetes mellitus (T2DM) is an expanding global health problem (1). There are 347 million people worldwide with diabetes, and more than 80% of diabetes deaths occur in low- and middle-income countries. According to statistics, 9.7% of people in China have type 2 diabetes, which not only threatens health, but also reduces quality of life and life expectancy (2). The onset of T2DM is multifactorial due to the interplay of common variation in multiple genes and environmental factors, but the exact pathogenesis of T2DM remains unclear (3).

Before type 2 diabetes occurs, glucose control is altered, which is reflected by higher fasting glucose and/or higher post-prandial glucose. This phenomenon is called impaired glucose regulation (IGR) and IGR is regarded as pre-diabetic state that includes impaired fasting glucose (IFG) and/or impaired glucose tolerance (IGT) (4). High glucose may cause an adverse effect on insulin sensitivity and secretion, further developing into glucotoxicity (5). Previous studies have shown that 5–10% IGT individuals develop diabetes each year, although some revert to normal glucose tolerance (6).

Recent developments in the understanding of T2DM have been heightened by the potential relevance of dysfunctional zinc signaling in this disease. Zinc is an important element for insulin storage and secretion (7). Zinc transporter 8 (ZnT-8), a member of the zinc transporter family, has been shown to bind with insulin in beta cells to form a solid hexamer, which is stored in secretory vesicles (8). Zinc transporter solute carrier family 30 member 8 (*SLC30A8*) is located on chromosome 8q24.11. It encodes ZnT-8, which is highly expressed in pancreatic islets and beta cells. ZnT-8 transports zinc from the cytoplasm into insulin secretary vesicles. Some studies have shown that the polymorphisms of *SLC30A8* are associated with β-cell function and insulin release *in vivo* (9, 10) and *in vitro* (11).

The non-synonymous single-nucleotide polymorphism, rs13266634, of *SLC30A8* causes an amino acid change from arginine (R) to tryptophan (W) at position 325 (Arg325Trp). The association of rs13266634 polymorphism in IGR and T2DM has been demonstrated in different ethnic groups via GWAS (12–16). The major C allele of the rs13266634 polymorphism is strongly associated with Chinese IGR and T2DM patients. However, the results are contradictory, and not all variants associated with type 2 diabetes are related with impaired glucose. These different results might be due to racial and regional differences, and they may be due to the limitation of the number of patients per study. To reduce the influence of diverse genetic backgrounds, a meta-analysis based on the Chinese population was performed to assess the relationship between rs13266634 polymorphism and IGR/T2DM.

## Materials and methods

### Search strategy

We searched the PubMed, Embase, Web of Science, China National Knowledge Infrastructure (CNKI), Wan Fang and CBMD databases for articles published prior to January 2018. The searching languages contained both English and Chinese, and only published studies were considered. The search strategy was based on combination of “*SLC30A8*,” “rs13266634,” “polymorphism^*^,” “variant^*^,” “genotype^*^,” “diabetes,” “T2DM,” “impaired glucose regulation,” “IGR,” “Chinese,” and “China.” References of retrieved articles were also screened and selected.

### Inclusion and exclusion criteria

Studies included in the meta-analysis met all of the following criteria: (1) the association between *SLC30A8* rs13266634 polymorphism and T2DM/IGR; (2) Chinese population; (3) sufficient data about allele or genotype frequency in cases and controls; (4) providing the odds ratio (OR) and its 95% confidence interval (95%CI) of the polymorphism; and (5) and clear diagnosis of T2DM/IGR. Studies were excluded if genotype frequency data in the controls demonstrated departure from Hardy-Weinberg equilibrium (HWE). When the same data were included in more than one publication, only the most relevant articles with the largest data set were included in the final analysis.

### Data extraction

The following data elements from each study were extracted: name of first author, year of publication, region of the study population, ethnicity of Chinese population, source of control, genotype method, diagnostic criteria, risk allele (C allele) frequency, number of cases and controls, mean (or median) body mass index (BMI), percentage of men in cases and controls, and mean (or median) age. Data were independently extracted by two investigators who reached a consensus on all of the items. If there was a lack of genotype information, we contacted the corresponding author to obtain required data.

### Risk of bias assessment

The quality of studies was also independently assessed by two reviewers (DF and ZFF) based on a risk of bias assessment for genetic association study which was modified on the basis of both traditional epidemiologic considerations and genetic issues that were developed by Thakkinstian et al. (17, 18). The score was divided into five domains, including information bias, confounding bias, selective reporting of outcomes, population stratification, and Hardy-Weinberg equilibrium (HWE) in the control group. Quality scores of each study ranged from 0 (lowest) to 15 (highest). Studies with scores ≤10 were categorized into low quality, while those with scores >10 were considered as high quality. Disagreement between the two reviewers was solved by a senior reviewer (JCX).

### Statistical analysis

We used Stata 11.0 (College Station, TX, USA) for all statistical analyses. The HWE was examined in control groups by Fisher's Exact Test. If the study was found not to be in HWE with *P*-value <0.05, it was considered to be disequilibrium. Both per-allele and per-genotype approaches were performed to estimate the effect of the rs13266634 polymorphism on the risk of T2DM or IGR.

Per-allele analysis: The risk allele C frequency for rs13266634 was estimated for each study by reported data, and the overall prevalence and 95% confidence interval (95%CI) were estimated. Odds ratios (ORs), as well as 95%CI, were also estimated.

Per-genotype analysis: The genotype effect was also calculated if the genotype data could be extracted from the study. Three odds ratios were estimated: CC vs. TT (OR1), CT vs. TT (OR2), and CC vs. CT (OR3). If the main effect of the genotype was statistically significant, further comparisons of OR1, OR2 and OR3 were explored. These pairwise differences were used to indicate the most appropriate genetic model as follows: (1). If OR1 = OR3≠1 and OR2 = 1, a recessive model was suggested. (2). If OR1 = OR2 ≠ 1 and OR3 = 1, then a dominant model was suggested. (3). If OR2 = 1/OR3 ≠ 1 and OR1 = 1, then a complete overdominant model is suggested. (4). If OR1 > OR2 > 1 and OR1 > OR3 > 1 (or OR1 < OR2 < 1 and OR1 < OR3 < 1), a co-dominant model was suggested.

The estimation of the allele or genotype effect on T2DM was calculated by an OR and 95%CI. The *Z*-test was used to determine the statistical significance of the pooled OR, and its *P*-value was used to determine if the overall SNP effect was significant (α = 0.05). The Q test based on a Chi-square analysis was used to assess the existence of heterogeneity. *p* = 0.01 was selected as the boundary value of the judgment to minimize type 2 errors (19). The pooled OR with 95% CI was calculated using the random-effects model based on the DerSimonian and Laird method (20). The random-effects model was chosen a priori because it is considered as more conservative than the fixed-effects model, as it accounts for both within- and between-study heterogeneity (21). In addition, the degree of heterogeneity was quantified using *I*^2^ (*I*^2^ < 25%, no heterogeneity; 25% < *I*^2^ < 50%, moderate heterogeneity; 50% < *I*^2^ < 75%, large heterogeneity; and *I*^2^ > 75% extreme heterogeneity) (22). A random-effects model was selected if *I*^2^ was >50%. The cause of heterogeneity was explored by fitting covariates (i.e., age, body mass index, percentage of men, source of control, genotype method, or sample size) in a meta-regression if these data were available. A subgroup analysis was performed according to publication year, source of control, sample size, and quality scores.

A sensitive analysis with a single study being removed each time was performed to reflect the influence of the individual data set on the pooled OR. Publication bias was evaluated using Egger's linear regression asymmetry test and visual inspection of funnel plots, and the influence of potential publication bias on results was explored by using the Duval and Tweedie trim-and-fill procedure (23, 24). *P* < 0.05 was considered statistically significant in all analyses, except for the Egger test (*p* < 0.10) because of the low power of the test.

### Trial sequential analysis

Meta-analyses might result in type-I errors owing to an increased risk of random error when fewer patients are involved, and due to continuous significance testing when a cumulative meta-analysis is updated with new studies (25, 26). Therefore, to assess the risks of random errors, trial sequential analysis (TSA) was performed using Stata 11.0 software package (metacumbounds command), which combines information size estimation for meta-analysis (cumulated sample size of included trials) with an adjusted threshold for statistical significance in the cumulative meta-analysis. TSA was conducted with the intention to maintain an overall 5% risk of a type I error and a power of 80%. For the calculation of the required information size, an intervention effect of a 20% relative risk reduction (RRR) was anticipated using the control event proportion calculated from the actual meta-analyses.

## Results

### Studies included in the meta-analysis

**Figure 1:**
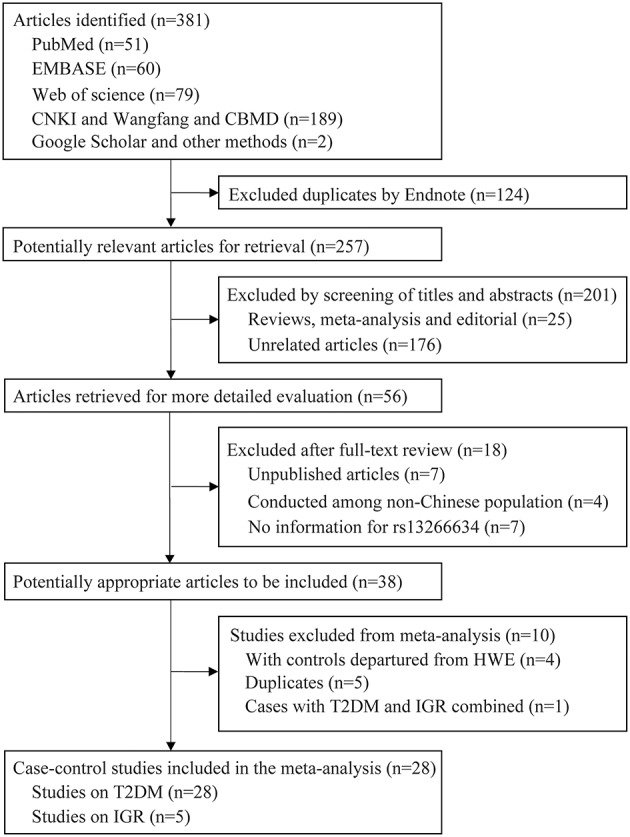
Flow chart for the selection of included studies.

A total of 381 articles were retrieved by literature search (Figure 1). After removal of 124 duplicates, 257 studies were screened for title and abstract as well as full text with 38 articles determined to be eligible. The following 10 studies were further excluded: five duplicate publications (27–31); four articles (32–35) with controls not in HWE; and one study (36) that reported data in combination with T2DM and IGR cases. Finally, 28 case-control studies with 25,912 cases and 26,975 controls were associated with T2DM, and five studies with 4,627 cases and 6,166 controls were associated with IGR. The characteristics and genotype distribution of included studies are listed in Table 1; Supplementary Table 1.

**Table 1:** The characteristics of the included studies in the meta-analysis.

**First author [ref]**	**Publication year**	**Region**	**Source of control**	**Genotype method**	**Diagnosis criteria**	**Sample size**		**Men (%)**		**Mean age (year)**		**BMI (kg/m**^**2**^**)**		**Quality score**
						**Case**	**Control**	**Case**	**Control**	**Case**	**Control**	**Case**	**Control**	
**T2DM**														
Wang (37)	2008	Chongqing	Hospital	PCR-RFLP	WHO	454	311	54.6	59.2	55	50	24.9	23.4	8
Wu (38)	2008	Beijing/shanghai	Population	SNPstream^*^	WHO	424	1,908	48.8	41.5	59.7	58.4	25.1	23.5	12
Xiang (39)	2008	Shanghai	Population	Mass array	WHO	521	721	40.3	37.4	62.6	59.7	26.3	24.1	13
Hu (40)	2009	Shanghai	Population	SNP-array	WHO	1,849	1,785	52.46	41.23	61.21	57.39	24.04	23.57	11
Han (41)	2010	Beijing	Population	SNapShot	WHO	992	993	52.7	34.2	56	58	25.0	25.0	11
Lin (42)	2010	Chengdu	Hospital	SNapShot	WHO	1,529	1,439	47.8	50	60.2	58.1	23.9	23.5	10
Shu (43)	2010	Shanghai	Population	SNP-array	SC	1,019	1,710	0	0	51.7	48.7	26.5	23.1	14
Tan (44)	2010	Singapore	Population	Mass array	WHO	1,541	2,196	NA	NA	NA	NA	NA	NA	12
Xu (45) CCS	2010	Shanghai	Population	SNapShot	SC	1,825	2,200	43.9	38.4	63.3	59.3	26.3	24.3	12
Li (46)	2011	Inner Mongolia	Hospital	AS-PCR	SC	125	97	54.4	55.67	57.90	52.55	25.72	23.99	7
Wang (47)	2011	Hengyang	Hospital	PCR-RFLP	WHO	236	218	51.3	50	57.4	54.1	23.8	22.4	6
Fu (48)	2012	Chongqing	Hospital	SNapShot	WHO	727	650	48	46.2	59.85	60.39	23.92	23.99	9
Zheng (49)	2012	Chongqing	Population	Mass array	WHO	227	152	61.67	38.82	54.05	52.80	25.27	23.67	7
Chen (50)	2013	Ningde	Population	SNP-assay	WHO	443	1,119	0	0	NA	NA	NA	NA	12
Tam (51)	2013	Hong Kong	Population	Mass array	WHO	5,882	2,569	45.5	47.5	56.8	41.8	25.1	21.8	12
Chang (52)	2014	TaiWan	Hospital + Population	SNPstream^*^	ADA	1,502	1,518	51.26	50.86	60.42	55.83	25.45	24.27	9
Chen (53)	2014	Gansu	Hospital	PCR-RFLP	WHO	116	80	51.72	51.25	50.99	46.99	24.7	24.4	5
Jin (54)	2014	Yanbian	Hospital	SNaPshot	WHO	313	178	52.2	39.8	61.15	49.18	23.86	25.3	6
Zhang (55)	2014	Gansu	Hospital	PCR-RFLP	WHO	123	129	56.91	57.36	49.76	46.98	NA	NA	6
Chen (56)	2015	ChangChun	Hospital	LDR	WHO	113	107	NA	NA	52.26	NA	25.59	NA	6
Kamila (57)	2015	Xinjiang	Hospital	PCR-RFLP	WHO	116	126	55.17	49.21	54.65	54.44	26.75	26.11	6
Liu (58)	2015	Jinzhou	Hospital	PCR-HRM	WHO	136	145	47.8	47.6	51.42	52.63	NA	NA	6
Qian (59)	2015	Jiangsu	Population	Mass array	ADA	2,925	3,281	37.5	37.6	58.21	56.57^a^	25.05	22.12	13
Su (60)	2015	Xinjiang	Hospital	Mass array	ADA	1,000	1,010	62.70	62.87	51.14	50.33	NA	NA	10
Zhang (61)	2015	Gansu	Hospital	PCR-RFLP	WHO	138	135	52.17	55.56	56.99	54.76	24.66	23.82	6
Zhang (61)	2015	Gansu	Hospital	PCR-RFLP	WHO	125	127	51.20	62.20	53.55	54.57	24.69	24.05	6
Zhao (62)	2015	Nanjing	Population	Mass array	WHO	1,737	1,950	42.95	31.33	64.31	57.69	25.2	23.59	12
Zou (63)	2016	Jilin	Hospital	PCR sequencing	WHO	214	243	60.54	48.97	41.6	22.5	25.6	21.5	6
**IGR**														
Wu (38)	2008	Beijing/shanghai	Population	SNPstream^*^	SC	878	1,908	48.4	41.5	58.6	58.4	25.2	23.5	12
Xiang (39)	2008	Shanghai	Population	Mass array	SC	375	721	37.3	37.4	63.7	59.7	25.7	24.1	13
Xu (45)	2010	Shanghai	Hospital	SNapShot	SC	1,487	2,200	40.0	38.4	61.0	59.3	25.5	24.3	12
Wang (47)	2011	Hengyang	Hospital	PCR-RFLP	SC	120	218	47.5	50	53.9	54.1	23.1	22.4	6
Chen (50)	2013	Ningde	Population	SNP-Assay	SC	1,767	1,119	0	0	NA	NA	NA	NA	12

* *Genome Lab SNPstream genotyping system*.

*ADA, American Diabetes Association; IGR, impaired fasting glucose; LDR, ligase detection reaction; NA, not available; PCR-HRM, polymerase chain reaction–high resolution melt; PCR-RFLP, cleaved amplification polymorphism sequence-tagged sites; SC, some diagnostic criteria but not clearly describe according to WHO criteria; SNP, single nucleotide polymorphisms; T2DM, type 2 diabetes mellitus; WHO, World Health Organization*.

### Association between SLC30A8 rs13266634 polymorphism and T2DM risk

**Figure 2:**
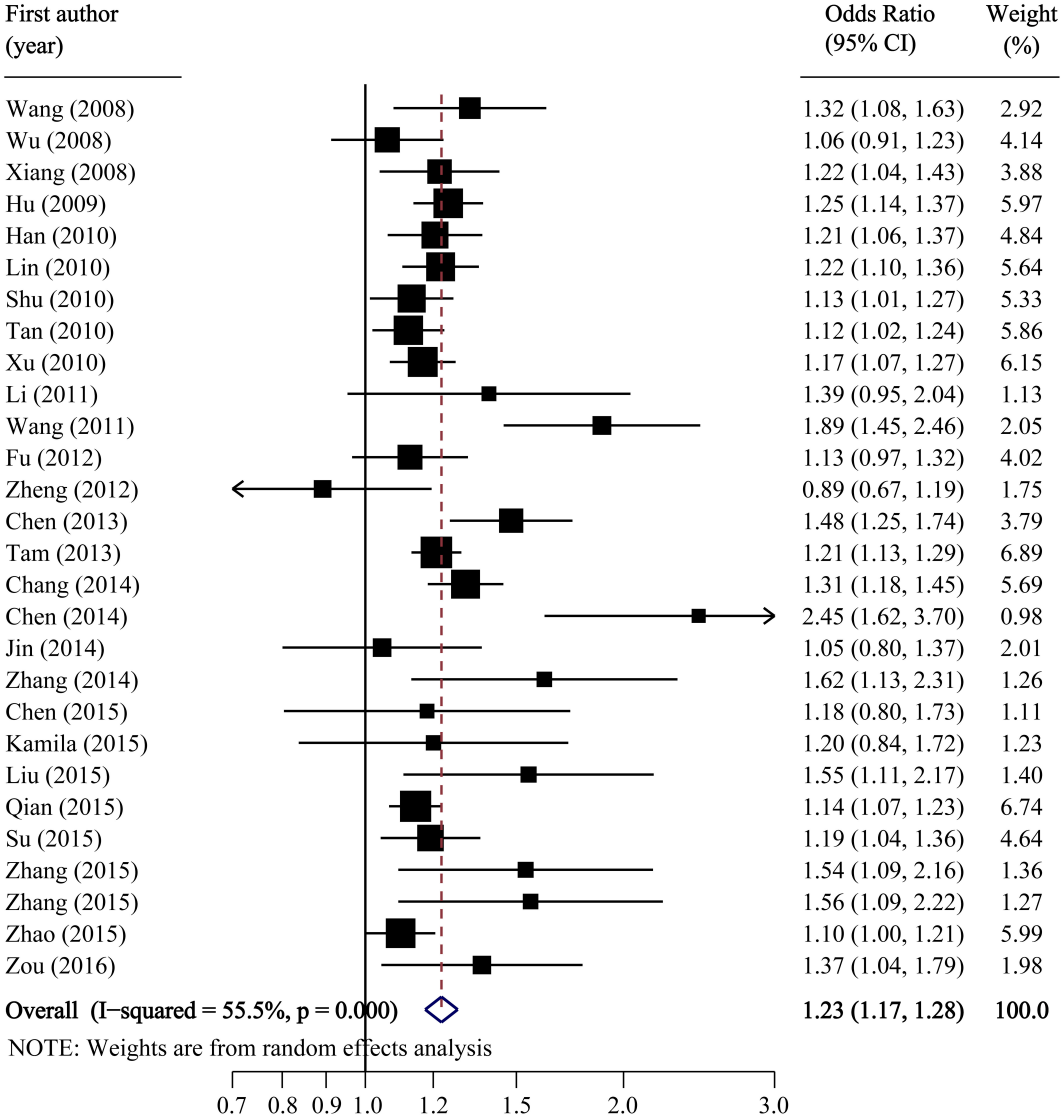
Forest plot of association between SLC30A8 rs13266634 polymorphism and T2DM risk in C vs. T model.

Per-allele analysis: The pooled allele frequency was calculated in both case and control groups. The risk allele C frequency was 60.6% (95%CI: 59.2–62.0%) in the T2DM group with high heterogeneity (*I*^2^ = 88.8%, *p* < 0.001) and 54.8% (95%CI: 53.2–56.4%) in the control group (*I*^2^ = 91.5%, *p* < 0.001). The odds ratio (C vs. T) was largely heterogenous (χ^2^ = 34.24, *p* = 0.008, *I*^2^ = 50.35) with a pooled odds ratio of 1.23 (95%CI: 1.17–1.28), and the overall SNP effect estimated by the random effect model was significant (*p* < 0.001; Table 2; Figure 2). These results suggested that individuals carrying the major C allele had 23% increased risk of developing T2DM than those carrying the minor T allele.

**Table 2:** Total and stratified analyses of SLC30A8 rs13266634 polymorphism and T2DM risk among Chinese.

**Summary**	***N***	**Cases/Controls**	**C vs. T**			**CC vs. TT**			**CT vs. TT**			**CC vs. CT**		
			**OR (95%CI)**	***P* for Z test**	***I*^2^, %**	**OR (95%CI)**	***P* for Z test**	***I*^2^, %**	**OR (95%CI)**	***P* for Z test**	***I*^2^, %**	**OR (95%CI)**	***P* for Z test**	***I*^2^, %**
Total	28	25,912/26,975	1.23 (1.17, 1.28)	<**0.001**	55.5	1.51 (1.38, 1.65)	<**0.001**	53.9	1.23 (1.15, 1.30)	<**0.001**	19.4	1.19 (1.14, 1.25)	<**0.001**	12.2
**PUBLICATION YEAR**														
< 2014	15	17,360/17,950	1.21 (1.15, 1.27)	<**0.001**	53.9	1.47 (1.32, 1.62)	<**0.001**	53.2	1.23 (1.14, 1.33)	<**0.001**	31.6	1.19 (1.13, 1.25)	<**0.001**	0.0
≥2014	13	8,552/9,025	1.28 (1.17, 1.40)	<**0.001**	60.3	1.65 (1.38, 1.97)	<**0.001**	58.1	1.22 (1.11, 1.34)	<**0.001**	7.7	1.25 (1.12, 1.40)	<**0.001**	44.1
**SOURCE OF CONTROL**^a^														
Population	12	18,951/20,466	1.17 (1.12, 1.22)	<**0.001**	42.8	1.36 (1.26, 1.47)	<**0.001**	36.9	1.16 (1.10, 1.23)	<**0.001**	0.0	1.18 (1.12, 1.23)	<**0.001**	0.0
Hospital	15	5,406/4,991	1.35 (1.23, 1.49)	<**0.001**	53.8	1.88 (1.56, 2.27)	<**0.001**	47.1	1.45 (1.25, 1.68)	<**0.001**	25.0	1.25 (1.11, 1.41)	<**0.001**	32.2
**SAMPLE SIZE**														
< 1,000	13	2,431/2,044	1.40 (1.22, 1.60)	<**0.001**	58.4	1.99 (1.51, 2.63)	<**0.001**	56.4	1.48 (1.20, 1.83)	<**0.001**	35.8	1.33 (1.11, 1.58)	<**0.001**	35.4
≥1,000	15	23,481/24,931	1.19 (1.15, 1.23)	<**0.001**	32.4	1.41 (1.32, 1.51)	<**0.001**	29.2	1.19 (1.13, 1.25)	<**0.001**	0.0	1.18 (1.13, 1.23)	<**0.001**	0.0
**SCORE**														
< 10	15	4,659/4,212	1.35 (1.22, 1.51)	<**0.001**	58.6	1.87 (1.51, 2.31)	<**0.001**	55.0	1.42 (1.22, 1.67)	<**0.001**	30.1	1.28 (1.12, 1.46)	<**0.001**	34.4
≥10	13	21,253/22,763	1.18 (1.14, 1.22)	<**0.001**	27.1	1.39 (1.30, 1.49)	<**0.001**	24.5	1.18 (1.11, 1.24)	<**0.001**	0.0	1.18 (1.13, 1.23)	<**0.001**	0.0
**SENSITIVITY ANALYSIS**														
Minimal	27	-/-	1.21 (1.16, 1.26) (47)	<**0.001**	47.2	1.48 (1.36, 1.60) (47)	<**0.001**	45.9	1.20 (1.15, 1.26) (47)	<**0.001**	0.0	1.18 (1.13, 1.24) (50)	<**0.001**	5.5
Maximal	27	-/-	1.24 (1.18, 1.29) (62)	<**0.001**	54.3	1.54 (1.40, 1.68) (62)	<**0.001**	52.8	1.24 (1.16, 1.32) (59)	<**0.001**	17.4	1.20 (1.15, 1.26) (62)	<**0.001**	11.0

a *One study with mixed of hospital and population source of control (52)*.

*OR, odds ratio; T2DM, type 2 diabetes mellitus; significant P-values in bold*.

The pooled OR and 95% CI for the association between the rs13266634 polymorphism and T2DM risk was 1.51 (1.38, 1.65; *p* < 0.001) with large between-study heterogeneity (*I*^2^ = 53.9%) for CC vs. TT, 1.23 (1.15, 1.30; *p* < 0.001) with small heterogeneity (*I*^2^ = 19.4%) for CT vs. TT, and 1.19 (1.14, 1.25; *p* < 0.001) with small heterogeneity (*I*^2^ = 12.2%) for CC vs. CT, which suggests a co-dominant model (CC vs. CT vs. TT) for the putative susceptibility allele C with T2DM (Table 2).

Stratified analysis indicated significantly stronger associations among controls coming from hospitals, among studies with lower number of participants, and among studies with lower quality for C vs. T (OR for controls from hospitals vs. populations: 1.35 vs. 1.17, *p* interaction = 0.008; OR for studies with sample size < 1,000 vs. ≥1,000: 1.40 vs. 1.19, *p* interaction = 0.023; OR for studies with quality score < 10 vs. ≥10: 1.35 vs. 1.18, *p* interaction = 0.018), for CC vs. TT (OR for controls from hospitals vs. populations: 1.88 vs. 1.36, *p* interaction = 0.002; OR for studies with sample size < 1,000 vs. ≥1,000: 1.51 vs. 1.32, *p* interaction = 0.018; OR for studies with quality score < 10 vs. ≥10: 1.51 vs. 1.30, *p* interaction = 0.009), and for CC vs. CT (OR for controls from hospitals vs. populations: 1.45 vs. 1.16, *p* interaction = 0.006; OR for studies with sample size < 1,000 vs. ≥1,000: 1.48 vs. 1.19, *p* interaction = 0.049; OR for studies with quality score < 10 vs. ≥10: 1.42 vs. 1.18, *p* interaction = 0.029; Table 2). Meta-regression further confirmed the effect of the source of control and quality score, but not total sample size, for C vs. T, CC vs. TT, and CC vs. CT comparisons (Supplementary Table 2). Influence analyses by removing one study each time revealed that the pooled ORs remained significant for all comparisons (Table 2).

**Figure 3:**
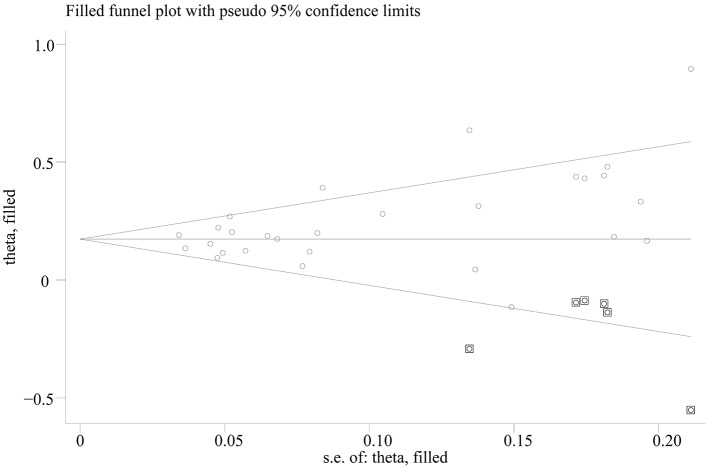
Trill and fill plot of associations between SLC30A8 rs13266634 polymorphism and T2DM risk in C vs. T model.

Egger's test showed significant evidence of publication bias (C vs. T: *p* = 0.014; CC vs. TT: *p* = 0.008; CC vs. TT: *p* = 0.015; CC vs. TT: *p* = 0.090), and the funnel plots for all comparisons were asymmetric. However, after imputing 6, 6, 4, and 1 missing studies for C vs. T, CC vs. TT, CC vs. TT, and CC vs. TT, respectively, by using the trim-and-fill method, the recalculated pooled ORs were not substantially different from the initial (Figure 3; Supplementary Table 3).

**Figure 4:**
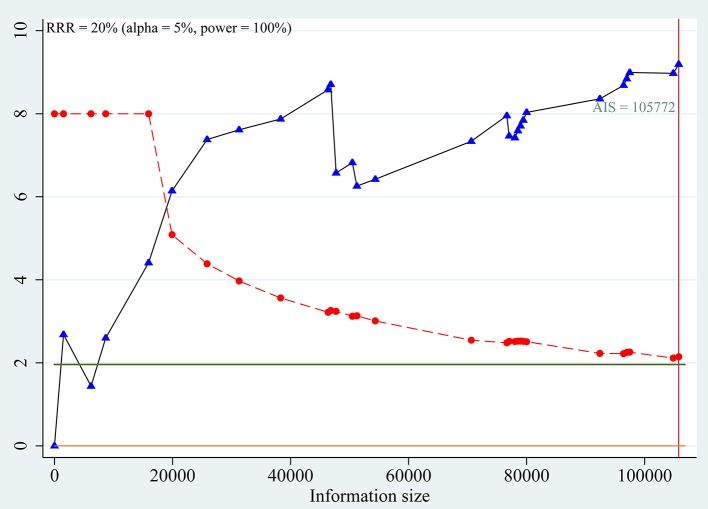
Trial sequential analysis for association between SLC30A8 rs13266634 polymorphism and T2DM risk in C vs. T model.

Trial sequential analyses: Because both the monitoring boundaries and information size had a cumulative Z-statistic >1.96, the evidence confirmed a risk effect of C allele on prevalence of T2DM (Figure 4).

### Association between SLC30A8 rs13266634 polymorphism and IGR risk

**Figure 5:**
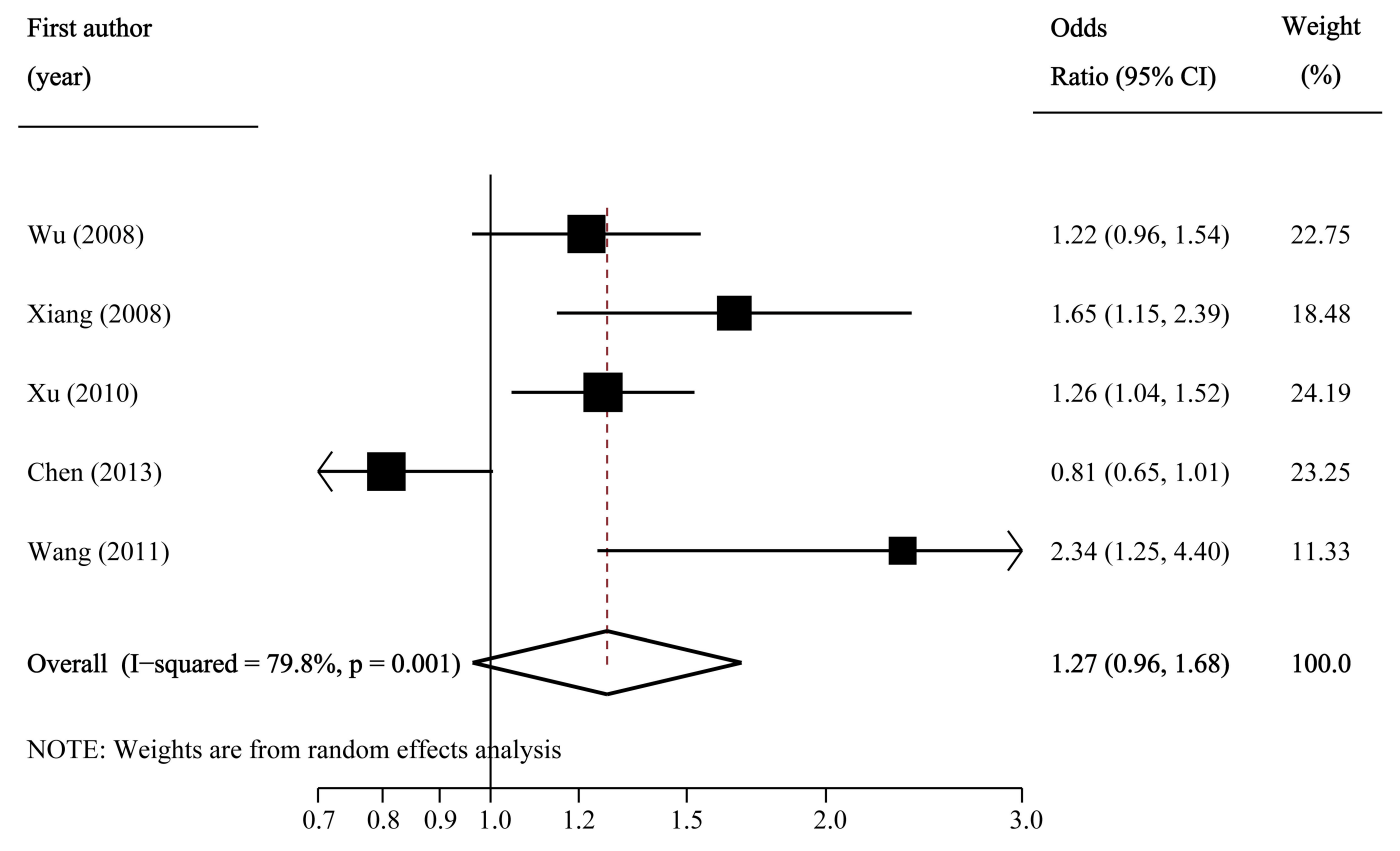
Forest plot of association between SLC30A8 rs13266634 polymorphism and IGR risk in C vs. T model.

The pooled frequency of the major C allele was estimated to be 57.2% (95%CI: 55.8–58.7%) with moderate heterogeneity (*I*^2^ = 43.0%) in IGR group, and it was 54.0% (95%CI: 50.5–57.5%; *I*^2^ = 90.0%) in the control group. The pooled OR of C vs. T was 1.13 (95% CI: 0.98–1.30; *p* = 0.082) with significant between-study heterogeneity (*I*^2^ = 81.0%; Figure 5). Egger's test (*p* = 0.257) suggested that there was no publication bias.

Per-genotype analysis also indicated that there were no significant associations between rs13266634 polymorphism and IGR risk (OR1: 1.27, 0.96–1.68, *p* = 0.089; OR2: 1.13, 0.95–1.34, *p* = 0.169; OR3: 1.08, 0.95–1.21, *p* = 0.243; Table 2).

**Figure 6:**
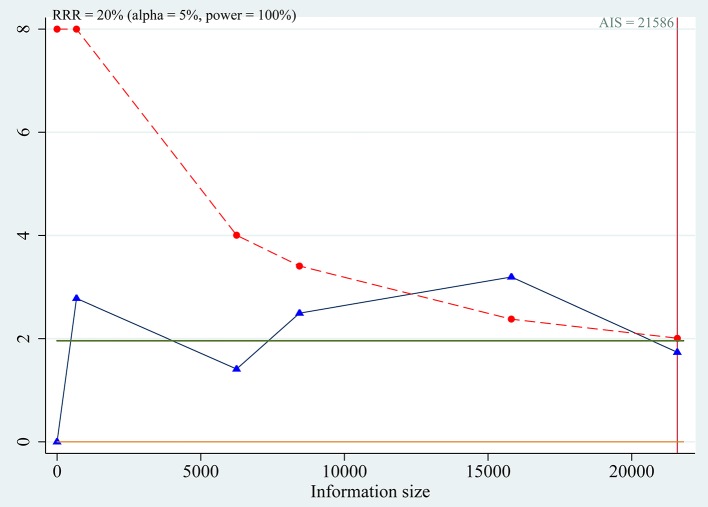
Trial sequential analysis for association between SLC30A8 rs13266634 polymorphism and IGR risk in C vs. T model.

Trial sequential analyses: because the cumulative z-curve fluctuated around both the traditional boundary and the trial sequential monitoring boundary, the evidence was not conclusive for the outcome (Figure 6).

## Discussion

A systematic review and meta-analysis was performed to investigate the association between *SLC30A8* rs13266634 and T2DM and IGR in a Chinese population, including 25,912 cases and 26,975 controls from 28 studies associated with T2DM, as well as 4,627 cases and 6,166 controls from five studies associated with IGR. The results suggested a significant association between the rs13266634 polymorphism and T2DM, which is consistent with previous results (64) but different from a study in Arab ethnicity (65). This association was also reported as significant in an African population under the allelic model, but neither under the codominant or recessive model (66). The expression differences of the same polymorphism between different ethnic groups might be caused by different genetic backgrounds and various environmental factors (67). In the present meta-analysis, individuals who carried the C allele had 23% increased risk of developing T2DM relative to those carrying the T allele in the Chinese ethnicity. These results were consistent but slightly higher than the GWAS database, which reports that people carrying the C allele may have an increased 18% risk of developing T2DM in a Finnish population (13). The genotype effect calculation showed that people with homozygous and heterozygous genotypes had 51 and 23%, respectively, higher risk of developing T2DM. The result agreed with GWAS research showing that homozygous and heterozygous individuals had 53 and 18%, respectively, higher risk of having T2DM (15). The frequency of risk allele C was 54.8% in the healthy Chinese group, which was lower than that in French (69.9%), Austrian (74.03%), and African American populations (91.59%) (15, 68). There were five case-control studies that described the association between the rs13266634 polymorphism and IGR, which included 4,627 cases and 6,166 controls. The evaluation indicated that the rs13266634 polymorphism was not associated with IGR in the Chinese population (OR = 1.13, 95%CI = 0.98–1.30, *p* = 0.082). It should be noted that this result was not consistent with the study in Europeans (13). These discrepancies may be attributed to the difference between genetic backgrounds of population substructure and sample size.

Zinc is necessary in β-cells for insulin crystallization in hexamers. Zinc is co-secreted with insulin, and participates in the regulation of β-cell mass by antioxidant actions (69, 70). Zinc plays an important role in β-cell function and insulin homeostasis. The ZnT8 transporter is primarily expressed in β-cells and co-localizes with insulin-containing secretory granules (71), and the alteration of ZnT8 expression may modulate insulin secretion. A previously study suggested that the *SLC30A8* rs13266634 polymorphism impairs ZnT8 expression in islets by disrupting the protein kinase A and protein kinase C recognition motif (R-X-S/T) in the ZnT8 molecule (7). Recent research has shown that the *SLC30A8* variant may affect glucose via modulating total zinc intake (72). These studies provided information for the underlying mechanisms of impaired glucose regulation and T2DM, potentially aiding the development of novel and individualized medical therapies. However, neither environmental triggers nor genetics alone can explain type 2 diabetes as a multifactorial disease. Thus, a close interaction between genetics and environment is presumed. Hence, it is still too early to draw such a conclusion until more functional research and larger population-based validation tests are performed.

Stratified analysis of control source, sample size, and quality score for T2DM showed that a significantly higher risk of T2DM was found in studies conducted using control source from hospitals with sample size <1,000 and with quality score <10. These results suggested that studies using controls from hospitals, small sample size, and low quality tend to overestimate the overall effect. However, subgroup analyses revealed that the risk effect of C allele from studies with controls from population, large sample size, and higher quality persisted. In addition, after excluding studies with controls from hospitals, small sample size, and especially low quality studies, the significant heterogeneity for allele comparison was markedly reduced from 55.5 to 27.1–42.8% for the CC vs. TT comparison as well as from 53.9 to 24.5–36.9% for the C vs. T comparison. The effect of quality score was the most evident because calculation of quality scores considered both the source of controls and sample size.

To the best of our knowledge, this is one of the most comprehensive meta-analysis for the association of *SLC30A8* rs13266634 polymorphism in IGR and T2DM risk in a Chinese population. A detailed search strategy was used in multiple databases, which was applied to include as many eligible studies as possible. Data extraction was performed in duplicate, and the qualities of included studies were evaluated by similar scale. Previous studies have confirmed the association between *SLC30A8* and T2DM (64, 73), but subgroup analysis was based only on different continents. Thus, it was necessary to perform a meta-analysis in the Chinese population due to its large population base and complicated genetic backgrounds. In addition, TSA was conducted to test if sufficient information size had been reached, minimizing potentially false positive results and providing the basis for further studies. TSA indicated that the cumulative *Z*-curve of the IGR fluctuated around both the traditional boundary and the trial sequential monitoring boundary, suggesting that additional studies are needed for this endpoint.

### Limitations

There were limitations in our study. First, case-control studies may overestimate the effect size of the association, which make the relationship between exposure and outcome less clear. To avoid such bias, the population should be based on a nested case-control study. Second, there were 19 articles with genotype data (19/28), and the estimation of genotype effects on T2DM may not be strong enough. Thus, a more precise association should be explored with sufficient data. These results should be interpreted with caution until further verification of sequencing approaches and larger meta-analysis are performed. Finally, significant publication bias was observed for all comparisons for T2DM. However, after imputing missing studies by using the trim-and-fill method, the recalculated pooled ORs were not substantially changed.

## Conclusion

The present meta-analysis indicated that the rs13266634 C allele in the *SLC30A8* gene was associated with T2DM risk in the Chinese population. More studies are needed to confirm the association between rs13266634 polymorphism and IGR risk. The current evidence is insufficient to inform clinical decision making or policies until better understanding of the full functional implications is obtained. In addition, comprehensive analyses of gene-gene and gene-environment interactions should also be evaluated in the future.

## Author contributions

CJ, GY, and FZ designed the study. FD and BZ extracted the data. SZ, XH, XD, and KZ performed the analyses. FD and FZ wrote the draft. CJ, GY, FZ, XC, JW, DL, ZW, XZ, YL, and SD revised it.

### Conflict of interest statement

The authors declare that the research was conducted in the absence of any commercial or financial relationships that could be construed as a potential conflict of interest.

## Abbreviations

95%CI: 95% confidence interval
BMI: body mass index
CNKI: China National Knowledge Infrastructure
GWAS: genome-wide association study
HWE: Hardy-Weinberg equilibrium
IFG: impaired fasting glucose
IGR: impaired glucose regulation
IGT: impaired glucose tolerance
LDR: ligase detection reaction
OR: odds ratio
PCR-HRM: polymerase chain reaction–high resolution melt
PCR-RFLP: cleaved amplification polymorphism sequence-tagged sites
RRR: relative risk reduction
SLC30A8: zinc transporter solute carrier family 30 member 8 gene
SNP: single nucleotide polymorphism
T2DM: type 2 diabetes mellitus
TSA: trial sequential analysis
WHO: World Health Organization
ZnT-8: zinc transporter 8
