# Supplementary material for: Association Between SLC30A8 rs13266634 Polymorphism and Risk of T2DM and IGR in Chinese Population: A Systematic Review and Meta-Analysis
Source: Front Endocrinol (Lausanne). 2018 Sep 25;9:564. doi: 10.3389/fendo.2018.00564 (PMC6167413; doi:10.3389/fendo.2018.00564)
Supplement: Supplementary file 1 [file Table_1.DOCX]

**Supplementary table 1** SLC30A8 rs13266634 polymorphism genotype distributions among T2DM/IGR cases and controls of the included studies

|  | Case | | |  | Control | | | T allele frequency (control) |
| --- | --- | --- | --- | --- | --- | --- | --- | --- |
|  | CC | CT | TT |  | CC | CT | TT |  |
| **T2DM** |  |  |  |  |  |  |  |  |
| Wang 2008 ([1](#_ENREF_1)) | 152 | 219 | 83 |  | 87 | 141 | 83 | 0.494 |
| Wu 2008 ([2](#_ENREF_2)) | 144 | 206 | 74 |  | 616 | 936 | 356 | 0.432 |
| Xiang 2008 ([3](#_ENREF_3)) | 175 | 254 | 92 |  | 203 | 359 | 159 | 0.469 |
| Hu 2009 ([4](#_ENREF_4)) | 695 | 877 | 277 |  | 558 | 880 | 347 | 0.441 |
| Han 2010 ([5](#_ENREF_5)) | 386 | 457 | 149 |  | 327 | 487 | 179 | 0.425 |
| [Lin](file:///C:\Users\Administrator\Desktop\张鲍欢--2018119\数据.xlsx#RANGE!_ENREF_25) ([6](#_ENREF_6)) | 532 | 740 | 257 |  | 420 | 715 | 304 | 0.460 |
| Shu 2010([7](#_ENREF_7)) | 379 | 485 | 155 |  | 572 | 829 | 300 | 0.420 |
| Tan ([8](#_ENREF_8)) | 433 | 670 | 259 |  | 617 | 1071 | 465 | 0.465 |
| Xu 2010 ([9](#_ENREF_9)) | 593 | 895 | 337 |  | 623 | 1095 | 482 | 0.468 |
| Li 2011([10](#_ENREF_10)) | 36 | 81 | 8 |  | 24 | 55 | 18 | 0.469 |
| Wang 2011([11](#_ENREF_11)) | 82 | 117 | 37 |  | 48 | 95 | 75 | 0.562 |
| Fu 2012([12](#_ENREF_12)) | 301 | 341 | 85 |  | 253 | 301 | 96 | 0.379 |
| Zheng 2012([13](#_ENREF_13)) | 65 | 114 | 48 |  | 48 | 76 | 28 | 0.434 |
| Chen 2013([14](#_ENREF_14)) | 205 | 193 | 45 |  | 390 | 541 | 188 | 0.410 |
| Tam ([15](#_ENREF_15)) | 1898 | 2740 | 989 |  | 713 | 1246 | 544 | 0.466 |
| Chang 2014([16](#_ENREF_16)) | 495 | 734 | 272 |  | 391 | 759 | 368 | 0.492 |
| Chen 2014([17](#_ENREF_17)) | 41 | 56 | 19 |  | 11 | 38 | 31 | 0.625 |
| Jin 2014([18](#_ENREF_18)) | 117 | 156 | 40 |  | 63 | 92 | 23 | 0.388 |
| Zhang 2014([19](#_ENREF_19)) | 48 | 56 | 19 |  | 30 | 65 | 30 | 0.500 |
| Chen 2015([20](#_ENREF_20)) | 44 | 55 | 14 |  | 36 | 55 | 16 | 0.407 |
| Kamila 2015([21](#_ENREF_21)) | 34 | 54 | 23 |  | 37 | 53 | 36 | 0.496 |
| Liu 2015([22](#_ENREF_22)) | 52 | 64 | 20 |  | 32 | 84 | 29 | 0.490 |
| Qian 2015([23](#_ENREF_23)) | 1044 | 1353 | 528 |  | 1038 | 1567 | 676 | 0.445 |
| Su 2015([24](#_ENREF_24)) | 484 | 445 | 71 |  | 449 | 452 | 108 | 0.331 |
| Zhang 2015^a^ ([25](#_ENREF_25)) | 56 | 62 | 20 |  | 33 | 76 | 26 | 0.474 |
| Zhang 2015^b^ ([25](#_ENREF_25)) | 51 | 54 | 20 |  | 33 | 65 | 29 | 0.484 |
| Zhao 2015([26](#_ENREF_26)) | 623 | 837 | 277 |  | 649 | 951 | 350 | 0.423 |
| Zou 2016([27](#_ENREF_27)) | 93 | 98 | 23 |  | 92 | 103 | 48 | 0.409 |
| **IGR** |  |  |  |  |  |  |  |  |
| Wu 2008 ([2](#_ENREF_2)) | 308 | 424 | 146 |  | 616 | 936 | 356 | 0.432 |
| Xiang 2008 ([3](#_ENREF_3)) | 131 | 181 | 62 |  | 203 | 359 | 159 | 0.470 |
| Xu 2010 ([9](#_ENREF_9)) | 468 | 732 | 287 |  | 623 | 1095 | 482 | 0.468 |
| Wang 2011([11](#_ENREF_11)) | 36 | 60 | 24 |  | 48 | 95 | 75 | 0.562 |
| Chen 2013([14](#_ENREF_14)) | 562 | 869 | 336 |  | 390 | 541 | 188 | 0.410 |

IGR: impaired fasting glucose; T2DM: type 2 diabetes mellitus.

**References**

1. Wang ZH, Zhang SH, Wang ZC, Gong LL, Li R, Ren W, et al. Relationship of rs12366634 polymorphism in SLA30A8 (solute carrier family, member 8) gene with type 2 diabetes in Chinese Han population. *Shanghai Medcial Journal* (2008) 31:323-7.

2. Wu Y, Li H, Loos RJ, Yu Z, Ye X, Chen L, et al. Common variants in CDKAL1, CDKN2A/B, IGF2BP2, SLC30A8, and HHEX/IDE genes are associated with type 2 diabetes and impaired fasting glucose in a Chinese Han population. *Diabetes* (2008) 57:2834-42. doi: 10.2337/db08-0047

3. Xiang J, Li XY, Xu M, Hong J, Huang Y, Tan JR, et al. Zinc transporter-8 gene (SLC30A8) is associated with type 2 diabetes in Chinese. *J Clin Endocrinol Metab* (2008) 93:4107-12. doi: 10.1210/jc.2008-0161

4. Hu C, Zhang R, Wang C, Wang J, Ma X, Lu J, et al. PPARG, KCNJ11, CDKAL1, CDKN2A-CDKN2B, IDE-KIF11-HHEX, IGF2BP2 and SLC30A8 are associated with type 2 diabetes in a Chinese population. *PLoS One* (2009) 4:e7643. doi: 10.1371/journal.pone.0007643

5. Han X, Luo Y, Ren Q, Zhang X, Wang F, Sun X, et al. Implication of genetic variants near SLC30A8, HHEX, CDKAL1, CDKN2A/B, IGF2BP2, FTO, TCF2, KCNQ1, and WFS1 in type 2 diabetes in a Chinese population. *BMC Med Genet* (2010) 11:81. doi: 10.1186/1471-2350-11-81

6. Lin Y, Li P, Cai L, Zhang B, Tang X, Zhang X, et al. Association study of genetic variants in eight genes/loci with type 2 diabetes in a Han Chinese population. *BMC Med Genet* (2010) 11:97. doi: 10.1186/1471-2350-11-97

7. Shu XO, Long J, Cai Q, Qi L, Xiang YB, Cho YS, et al. Identification of new genetic risk variants for type 2 diabetes. *PLoS Genet* (2010) 6:e1001127. doi: 10.1371/journal.pgen.1001127

8. Tan JT, Ng DP, Nurbaya S, Ye S, Lim XL, Leong H, et al. Polymorphisms identified through genome-wide association studies and their associations with type 2 diabetes in Chinese, Malays, and Asian-Indians in Singapore. *J Clin Endocrinol Metab* (2010) 95:390-7. doi: 10.1210/jc.2009-0688

9. Xu M, Bi Y, Xu Y, Yu B, Huang Y, Gu L, et al. Combined effects of 19 common variations on type 2 diabetes in Chinese: results from two community-based studies. *PLoS One* (2010) 5:e14022. doi: 10.1371/journal.pone.0014022

10. Li XJ, Su Y, Yan CL, Zhang JJ, Gu L, Qin WB, et al. Association of rs13266634 Polymorphism in SLC30A8 Gene with Type 2 Diabetes in Han Population of Inner Mongolia. *Progress in Modern Biomedicine* (2011):2221-3

11. Wang JP, Li J, Deng SS. The relationship between polymorphisms of solute carrier family 30, member 8 gene and Type 2 Diabetes. *Chinese General Practice* (2011) 14:1296-9.

12. Fu LL, Lin Y, Yang ZL, Yin YB. Association analysis of genetic polymorphisms of TCF7L2, CDKAL1, SLC30A8, HHEX genes and microvascular complications of type 2 diabetes mellitus. *Zhong Hua Yi Xue Yi Chuan Xue Za Zhi* (2012) 29:194-9.

13. Zheng X, Ren W, Zhang S, Liu J, Li S, Li J, et al. Association of type 2 diabetes susceptibility genes (TCF7L2, SLC30A8, PCSK1 and PCSK2) and proinsulin conversion in a Chinese population. *Mol Biol Rep* (2012) 39:17-23. doi: 10.1007/s11033-011-0705-6

14. Chen G, Xu Y, Lin Y, Lai X, Yao J, Huang B, et al. Association study of genetic variants of 17 diabetes-related genes/loci and cardiovascular risk and diabetic nephropathy in the Chinese She population. *J Diabetes* (2013) 5:136-45. doi: 10.1111/1753-0407.12025

15. Tam CH, Ho JS, Wang Y, Lam VK, Lee HM, Jiang G, et al. Use of net reclassification improvement (NRI) method confirms the utility of combined genetic risk score to predict type 2 diabetes. *PLoS One* (2013) 8:e83093. doi: 10.1371/journal.pone.0083093

16. Chang YC, Liu PH, Yu YH, Kuo SS, Chang TJ, Jiang YD, et al. Validation of type 2 diabetes risk variants identified by genome-wide association studies in Han Chinese population: a replication study and meta-analysis. *PLoS One* (2014) 9:e95045. doi: 10.1371/journal.pone.0095045

17. Chen HQ, Liu J, Zhang Q, Liu JX, Zhao J, Wang YF, et al. Correlation between solute carrier family 30,member 8 gene polymorphism and type 2 diabetes mellitus in Gansu Han population. *J Lanzhou University* (2014):21-5.

18. Jin JJ, Gong KY, Zhou WJ. Correlation and interaction between minor genes and type 2 diabetes mellitus in Korean-Chinese. *J Medical Science Yanbian University* (2014):84-88.

19. Zhang SL, Liu J, Liu JX, Wei SH. Association of rs13266634 Polymorphism in SLC30A8 Gene with Type 2 Diabetes in Chinese Dongxiang Nationality. *Gansu Science and Technology* (2014) 30:138-139.

20. Chen Y, Zhao Y, Liu GF. Association of SLC30A8, CDKN2A/2B, HHEX and TCF7L2 gene polymorphisms with type 2 diabetes in northeastern China. *Chin J Gerontology* (2015):4201-4.

21. Bali K. Study on realtionship between the rs12366634 polymorphism of TCF7L2 gene and type 2 diabetes in Xinjiang Han populations. Xinjiang Medcial School, Master Thesis (2015).

22. Liu Y, Wang ZY, Chi ZH, Wang T. Research on Correlation between rs13266634 C/T SNP of SLC30A8 Gene and Susceptibility to Type 2 Diabetes Mellitus. *J China Medical University* (2015):494-7.

23. Qian Y, Lu F, Dong M, Lin Y, Li H, Dai J, et al. Cumulative effect and predictive value of genetic variants associated with type 2 diabetes in Han Chinese: a case-control study. *PLoS One* (2015) 10:e0116537. doi: 10.1371/journal.pone.0116537

24. Su YX, Wang ZQ, Yao H, Wang TT, Ma Q, Zhu J, et al. Association between type 2 diabetes in Uygur and polymorphisms of SLC30A8 and its interaction with smoking. *Chin J Epidemiol* (2015) 36:1167-71.

25. Zhang SL, Liu J, Liu JX, Liu J, Xu YJ, Wei SH. The research of relationship between SLC30A8 gene rs 13266634 C/T polymorphism and type 2 diabetes mellitus risk in the Dongxiang and Moselm nationality in Gansu. *Chin J Gerontology* (2015) 35: 898-900.

26. Zhao LJ, Xu KF, Yang T, Dai H, Ma BQ, Ji L, et al. Relationship between SLC30A8 and PTPRD gene polymorphisms and type 2 diabetes in middle aged and elderly people in Nanjing area. *Int J Endocrinol Metab* (2015) 35:145-8.

27. Zou J. Serum levels of specific autoantibodies in patients with autoimmune diabetes and thyroid disease and gene polymorphism correlation analysis. Jilin University, Master Thesis (2016).
