# Supplementary material for: Association Between SLC30A8 rs13266634 Polymorphism and Risk of T2DM and IGR in Chinese Population: A Systematic Review and Meta-Analysis
Source: Front Endocrinol (Lausanne). 2018 Sep 25;9:564. doi: 10.3389/fendo.2018.00564 (PMC6167413; doi:10.3389/fendo.2018.00564)
Supplement: Supplementary file 2 [file Table_2.DOCX]

**Supplementary table 2** Meta regression analyses for phenotype of T2DM

|  | coefficient | SE | t | P | Lower limit | Upper limit |
| --- | --- | --- | --- | --- | --- | --- |
| **C vs T** |  |  |  |  |  |  |
| Publication year | .0088467 | .0108265 | 0.82 | 0.421 | -.0134075 | .0311008 |
| Men% | .0010023 | .0019308 | 0.52 | 0.608 | -.0029828 | .0049873 |
| Age | -.0065704 | .005117 | -1.28 | 0.212 | -.0171558 | .0040149 |
| BMI | -.0169097 | .047792 | -0.35 | 0.727 | -.116602 | .0827826 |
| Source of control | .1164507 | .0521162 | 2.23 | **0.035** | .0091152 | .2237861 |
| Total sample | -.0000197 | .0000118 | -1.67 | 0.107 | -.0000439 | 4.57e-06 |
| Quality score | -.0268347 | .0086482 | -3.10 | **0.005** | -.0446115 | -.009058 |
| **CC vs. TT** |  |  |  |  |  |  |
| Publication year | .0189418 | .0215099 | 0.88 | 0.387 | -.0252724 | .0631561 |
| Men% | .0029862 | .0037964 | 0.79 | 0.439 | -.0048491 | .0108215 |
| Age | -.0160672 | .0103544 | -1.55 | 0.134 | -.0374869 | .0053526 |
| BMI | -.0310018 | .0947243 | -0.33 | 0.747 | -.2285932 | .1665896 |
| Source of control | .2579511 | .0964801 | 2.67 | **0.013** | .0592465 | .4566556 |
| Total sample | -.000041 | .000023 | -1.79 | 0.086 | -.0000882 | 6.16e-06 |
| Quality score | -.0593078 | .0170729 | -3.47 | **0.002** | -.0944016 | -.024214 |
| **CT vs. TT** |  |  |  |  |  |  |
| Publication year | .0002397 | .0116351 | 0.02 | 0.984 | -.0236766 | .024156 |
| Men% | .0028848 | .0022615 | 1.28 | 0.214 | -.0017827 | .0075523 |
| Age | -.0106445 | .0067642 | -1.57 | 0.129 | -.0246373 | .0033482 |
| BMI | .002974 | .0544505 | 0.05 | 0.957 | -.1106078 | .1165559 |
| Source of control | .1906744 | .064307 | 2.97 | **0.007** | .0582317 | .3231171 |
| Total sample | -.0000176 | .0000112 | -1.57 | 0.128 | -.0000406 | 5.43e-06 |
| Quality score | -.0403035 | .0128148 | -3.15 | **0.004** | -.0666448 | -.0139622 |
| **CC vs. CT** |  |  |  |  |  |  |
| Publication year | .0022463 | .0090049 | 0.25 | 0.805 | -.0162635 | .0207561 |
| Men% | -.0001688 | .0016373 | -0.10 | 0.919 | -.0035481 | .0032105 |
| Age | -.0007728 | .0050698 | -0.15 | 0.880 | -.0112604 | .0097148 |
| BMI | .0043251 | .0379031 | 0.11 | 0.910 | -.0747394 | .0833895 |
| Source of control | .0244879 | .0538932 | 0.45 | 0.653 | -.0865073 | .1354831 |
| Total sample | -4.12e-06 | 9.34e-06 | -0.44 | 0.663 | -.0000233 | .0000151 |
| Quality score | -.0166042 | .0109622 | -1.51 | 0.142 | -.0391373 | .0059288 |
