# Supplementary material for: Association Between SLC30A8 rs13266634 Polymorphism and Risk of T2DM and IGR in Chinese Population: A Systematic Review and Meta-Analysis
Source: Front Endocrinol (Lausanne). 2018 Sep 25;9:564. doi: 10.3389/fendo.2018.00564 (PMC6167413; doi:10.3389/fendo.2018.00564)
Supplement: Supplementary file 3 [file Table_3.DOCX]

**Supplementary table 3** Egger’s test and trim-and-fill analyses for T2DM

|  | P for Egger’s test | No. need to fill | OR (95% CIs)^a^ |
| --- | --- | --- | --- |
| C vs. T | 0.014 | 6 | 1.19 (1.13, 1.25) |
| CC vs. TT | 0.008 | 6 | 1.43 (1.29, 1.58) |
| CT vs. TT | 0.015 | 4 | 1.20 (1.11, 1.29) |
| CC vs. CT | 0.090 | 1 | 1.19 (1.14, 1.25) |

^a^ OR (95% CIs) after fill using trim-and-fill analyses.
